# Effects of night-time and weekend admissions on in-hospital mortality in acute myocardial infarction patients in Japan

**DOI:** 10.1371/journal.pone.0191460

**Published:** 2018-01-19

**Authors:** Seiko Mizuno, Susumu Kunisawa, Noriko Sasaki, Kiyohide Fushimi, Yuichi Imanaka

**Affiliations:** 1 Department of Healthcare Economics and Quality Management, Graduate School of Medicine, Kyoto University, Sakyo-ku, Japan; 2 Department of Health Policy and Informatics, Graduate School of Medicine, Tokyo Medical and Dental University, Yushima, Bunkyo-ku, Tokyo, Japan; University of Colorado Denver, UNITED STATES

## Abstract

**Background:**

Patients admitted to hospital during off-hours may experience poorer quality of care and clinical outcomes. However, few studies have examined the variations in clinical processes and outcomes across admission times and days of the week in acute myocardial infarction (AMI) patients. This study aimed to comparatively analyze the effect of weekend and weekday admissions stratified by admission time on in-hospital mortality in AMI patients.

**Methods and results:**

Using a large nationwide administrative database, we analyzed 103,908 AMI patients admitted to 639 Japanese acute care hospitals between April 2011 and March 2015. We divided patients into the following 4 groups: weekday daytime admissions, weekday night-time admissions, weekend daytime admissions, and weekend night-time admissions. A hierarchical logistic regression model was used to comparatively examine in-hospital mortality among the groups after adjusting for age, sex, ambulance use, Killip class, comorbidities, and the number of cardiologists in the admitting hospital. In addition, we also calculated and compared the adjusted odds ratios of various AMI therapies among the groups. The in-hospital mortality rate of weekend daytime admissions was higher than those admitted during other times (weekday daytime: 6.8%; weekday night-time; 6.5%, weekend daytime; 7.6%; weekend night-time: 6.6%; *P* < 0.001), even after adjusting for the covariates (adjusted odds ratio for weekend daytime admissions: 1.10; 95% confidence interval: 1.03–1.19). The prescription rates of guideline-based medications provided on the first day of admission were higher in night-time admissions than in daytime admissions.

**Conclusions:**

In-hospital mortality rates were higher in AMI patients admitted during weekend daytime hours when compared with patients admitted during other times. Furthermore, patients admitted during daytime hours had lower prescription rates of guideline-based medications. Our findings indicate that weekend daytime admissions may be a potential target for improvement in the Japanese healthcare system.

## Introduction

Previous studies have described an “off-hours effect” where patients admitted to hospital during off-hours experience poorer quality of care and clinical outcomes [1–3]. This relationship may be the result of time-dependent differences in the availability of healthcare services and staff [1,4], but there is little evidence of the mechanisms involved. Furthermore, these studies have generally focused on the differences between off-hour and regular hour admissions or between weekday and weekend admissions. However, few studies have examined the variations in clinical processes and outcomes across admission times and days of the week [2,5]. As healthcare services may fluctuate over these time periods [2], it is important to investigate the associated patterns of healthcare quality in order to identify potential areas for improvement.

Acute myocardial infarction (AMI) remains a leading cause of death [6] and a major contributor to global disease burden [7]. Despite the availability of well-established acute interventions for treating AMI patients [8,9], previous research has indicated that the delayed implementation of these interventions during weekends or off-hours can result in poorer outcomes [5,10,11]. The ability to provide the necessary care may vary not only according to the days of admission, but also the time of admission [5]. Many institutions need to call in off-duty staff to perform these specialized interventions during weekends and off-hours [12]. As there are more medical staff on duty during the day, we hypothesized that admissions during weekday daytime hours would have the best overall clinical outcomes, and that night-time admissions on weekends and weekdays would be associated with poorer clinical outcomes than admissions during weekend daytime hours.

In this study, we comparatively analyzed the effect of weekend and weekday admissions stratified by admission time on in-hospital mortality in AMI patients.

## Methods

### Data source

The Diagnosis Procedure Combination (DPC) system is a case-mix classification system used in Japan to calculate reimbursements from insurers to acute care hospitals. This study used the DPC database, which consists of administrative claims data regularly collected from voluntarily participating hospitals that operate under the DPC system. The collection of data is funded by Japan’s Ministry of Health, Labour and Welfare. These data are obtained from approximately 80% of all DPC hospitals, and encompass approximately 8 million inpatient episodes per year [13]. The DPC database includes summarized inpatient information, such as recorded diagnoses of the disease that resulted in hospitalization, other major diagnoses, Killip class on admission, comorbidities on admission, and discharge status. Diseases are identified through International Classification of Disease, 10th revision (ICD-10) codes. The database also contains detailed information on the use of medical resources, diagnostic tests, surgical procedures, and prescribed medications.

### Subject inclusion and exclusion criteria

We first identified candidate subjects who fulfilled the following criteria: 1) patients aged 18 years or older at the time of admission; 2) patients admitted with transmural AMI (ICD-10 codes: I21.1, I21.2, and I21.3) between April 1, 2011 and March 31, 2015; and 3) patients with Killip classification in their data. The following cases were excluded from analysis: 1) patients who were discharged alive within 2 days of admission; 2) patients who were hospitalized for more than 90 days; 3) patients with missing data for ambulance use, reason for admission, and clinical outcomes; and 4) patients whose comorbidities on admission included out-of-hospital cardiac arrest (ICD-10 codes: I46.0, I46.1, and I46.9). The analysis was limited to hospitals that had continuously provided DPC data for at least 4 years.

### Baseline variables

We analyzed patient age, sex, ambulance use, referrals from clinics, Killip class on admission, and comorbidities on admission. Relevant comorbidities were identified based on those reported in prior research [14–16]; these included fatal arrhythmia, atrial fibrillation/atrial flutter, hypertension, hyperlipidemia, cerebrovascular disease, diabetes mellitus, renal disease, chronic pulmonary disease, and old myocardial infarction. Age was grouped into 4 categories (≤60 years, 61–70 years, 71–80 years, and ≥81 years) for analysis.

Weekend admissions were defined as those that took place on Saturdays, Sundays, and Japanese national holidays; weekday admissions were defined as those that took place on Mondays to Fridays. Japanese national holidays included those specified in the Act on National Holidays [17] as well as the *de facto* New Year holidays (December 29–31 and January 2–3). Daytime hours were defined as the period from 6:00 AM to 9:59 PM, and night-time hours were defined as the period from 10:00 PM to 5:59 AM.

A list of certified cardiologists was obtained from the website of the Japanese Circulation Society [18], which certifies cardiologists who fulfill the stipulated education and practice criteria. We merged these data with the hospital dataset to identify the number of certified cardiologists in each hospital.

### Statistical analysis

We divided the study population into the following 4 groups according to each patient’s day and time of admission: weekday daytime admissions, weekday night-time admissions, weekend daytime admissions, and weekend night-time admissions.

The primary outcome measure used in this study was all-cause in-hospital mortality. In order to compare the quality of care among the 4 groups, we examined the differences in the implementation rates of the following procedures: percutaneous coronary intervention (PCI), intra-aortic balloon pumping, coronary artery bypass graft, and venoarterial extracorporeal membrane oxygenation. In addition, we also compared the prescription rates of guideline-based medications (aspirin, thienopyridine derivatives, renin-angiotensin system blockers, β-blockers, and hydroxymethylglutaryl-CoA reductase inhibitors [statins]) on the day of admission and throughout hospitalization. Baseline patient characteristics, medical procedures, and guideline-based medications were compared among the 4 groups using the chi-squared test and Kruskal-Wallis test, as appropriate. In-hospital mortality was analyzed using Fisher’s exact test.

Hierarchical logistic regression analysis of in-hospital mortality was conducted by fitting multilevel logistic regression models with hospital-level random intercepts. The independent variables included age, sex, Killip class on admission, ambulance use, comorbidities on admission, and number of cardiologists in the admitting hospital. We also investigated whether the rates of guideline-based procedures and medications differed among the 4 groups using hierarchical logistic regression analyses that adjusted for patient age, sex, and Killip class.

In all logistic regression analyses, we determined the odds ratios (ORs) and 95% confidence intervals (CIs) for each variable. A 2-sided significance level of 0.05 was used, and all analyses were conducted using R statistical software (version 3.4.0).

### Ethical considerations

This study was conducted in accordance with the Ethical Guidelines for Medical and Health Research involving Human Subjects issued by the Japanese national government. These guidelines include a stipulation for the protection of patient anonymity. As the data were anonymized, the requirement for informed consent was waived. This study was approved (Approval number: R0135) by the Ethics Committee, Kyoto University Graduate School and Faculty of Medicine.

## Results

### Patient characteristics

We identified a total of 126,100 AMI patients in the DPC database. We excluded patients who were discharged alive within 2 days of admission (n = 1,155), hospitalized for more than 90 days (n = 1,123), had missing data (n = 3,622), and had been diagnosed with out-of-hospital cardiac arrest (n = 827). After excluding hospitals that had not continuously provided DPC data for at least 4 years, our final sample comprised 103,908 patients admitted to 639 hospitals.

Table 1 summarizes the baseline characteristics of the patients. There was a higher proportion of men and ambulance use for night-time admissions than daytime admissions. The clinic referral rates were generally higher in daytime admissions than in night-time admissions. In addition, patients admitted during weekday daytime hours tended to have a lower Killip class than the other patients. Table 1 also shows the rates of guideline-based procedures and medications during the day of admission and throughout the hospitalization episode. More than 80% of patients underwent PCI on the day of admission in all 4 groups. A higher proportion of night-time admissions were administered aspirin on the day of admission relative to daytime admissions.

**Table 1:** Patient characteristics according to admission days and times.

No. of patients		Weekday daytime 67441	Weekday night-time 9098	Weekend daytime 22911	Weekend night-time 4458	*P*-value
Age, years, median (IQR)		77 (61–79)	66 (57–76)	69 (60–79)	66 (56–76)	<0.05
Male		49509 (73.4)	7016 (77.1)	17011 (74.2)	3482 (78.1)	<0.001
Ambulance use		40776 (60.5)	7023 (77.2)	15520 (67.7)	3456 (77.5)	<0.001
Clinic referral		30915 (45.8)	2128 (23.4)	7987 (34.9)	1044 (23.4)	<0.001
Killip classification on admission	1	34082 (50.5)	4415 (48.5)	11357 (49.6)	2173 (48.7)	<0.001
	2	19983 (29.6)	2602 (28.6)	6618 (28.9)	1250 (28.0)	
	3	5725 (8.5)	853 (9.4)	1970 (8.6)	412 (9.2)	
	4	7651 (11.3)	1228 (13.5)	2966 (12.9)	623 (14.0)	
Comorbidities present on admission						
Fatal arrhythmia		3042 (4.5)	456 (5.0)	1251 (5.5)	240 (5.4)	<0.001
Atrial fibrillation/Atrial flutter		3351 (5.0)	400 (4.4)	1165 (5.1)	197 (4.4)	0.024
Hypertension		43409 (64.4)	5943 (65.3)	14660 (64.0)	2977 (66.8)	0.001
Hyperlipidemia		40150 (59.5)	5719 (62.9)	13473 (58.8)	2866 (64.3)	<0.001
Cerebrovascular disease		3269 (4.8)	345 (3.8)	1061 (4.6)	158 (3.5)	<0.001
Diabetes		20201 (30.0)	2579 (28.3)	6554 (28.6)	1186 (26.6)	<0.001
Renal disease		2827 (4.2)	234 (2.6)	921 (4.0)	110 (2.5)	<0.001
Chronic pulmonary disease		1540 (2.3)	166 (1.8)	510 (2.2)	83 (1.9)	0.015
Old myocardial infarction		959 (1.4)	94 (1.0)	351 (1.5)	57 (1.3)	0.006
Revascularization therapy provided on the day of admission						
PCI		54052 (80.1)	7481 (82.2)	19055 (83.2)	3625 (81.3)	<0.001
CABG		438 (0.6)	59 (0.6)	113 (0.5)	22 (0.5)	0.044
Mechanical support provided on the day of admission						
IABP		8010 (11.9)	1286 (14.1)	2975 (13.0)	623 (14.0)	<0.001
ECMO		825 (1.2)	119 (1.3)	325 (1.4)	66 (1.5)	0.085
Drugs administered on the day of admission						
Aspirin		47695 (70.7)	7078 (77.8)	16357 (71.4)	3496 (78.4)	<0.001
ACE-I/ARB		15463 (22.9)	2991 (32.9)	5393 (23.5)	1501 (33.7)	<0.001
Statins		25445 (37.7)	4354 (47.9)	8508 (37.1)	2115 (47.4)	<0.001
β-blockers		10685 (15.8)	1897 (20.9)	3572 (15.6)	920 (20.6)	<0.001
In-hospital mortality		4566 (6.8)	594 (6.5)	1742 (7.6)	294 (6.6)	<0.001

Values are presented as n (%) unless otherwise stated. Abbreviations: ACE-I: angiotensin converting enzyme-inhibitors, ARB: angiotensin II receptor blockers; CABG, coronary artery bypass graft; ECMO, extracorporeal membrane oxygenation; IABP, intra-aortic balloon pumping; IQR, interquartile range, PCI, percutaneous coronary intervention.

Table 2 shows the results of the logistic regression analysis of guideline-based procedures and medications after adjusting for age, sex, and Killip class. Patients admitted during weekend daytime hours were more likely to undergo PCI. In addition, the prescription rates of other guideline-based medications on the day of admission were higher during night-time admissions after adjusting for the covariates.

**Table 2:** Adjusted odds ratios (95% confidence intervals) of guideline-based procedures and medications for acute myocardial infarction patients according to admission days and times.

Variables	Weekday daytime n = 67441	Weekday night-time n = 9098	Weekend daytime n = 22911	Weekend night-time n = 4458
Revascularization therapy provided on the day of admission				
PCI	Ref.	1.05 (0.99–1.11)	1.21 (1.16–1.26)	0.96 (0.89–1.04)
CABG	Ref.	0.92 (0.69–1.22)	0.73 (0.59–0.91)	0.61 (0.39–0.95)
Mechanical support provided on the day of admission				
IABP	Ref.	1.08 (1.01–1.16)	1.04 (0.99–1.09)	1.02 (0.92–1.13)
ECMO	Ref.	0.80 (0.65–0.99)	1.02 (0.88–1.17)	0.90 (0.68–1.18)
Drugs administered on the day of admission				
Aspirin	Ref.	1.45 (1.37–1.53)	1.04 (1.00–1.08)	1.47 (1.36–1.59)
ACE-I/ARB	Ref.	1.75 (1.66–1.84)	1.04 (1.00–1.08)	1.80 (1.67–1.93)
Statins	Ref.	1.57 (1.49–1.64)	0.97 (0.94–1.00)	1.50 (1.41–1.61)
β-blockers	Ref.	1.46 (1.38–1.55)	0.99 (0.94–1.03)	1.40 (1.29–1.52)

ORs were adjusted for sex, age-group (≤60 years, 61–70 years, 71–80 years, and ≥81 years), and Killip classification using multivariable regression analysis. Abbreviations: ACE-I: angiotensin converting enzyme-inhibitors, ARB: angiotensin II receptor blockers; CABG, coronary artery bypass graft; ECMO, extracorporeal membrane oxygenation; IABP, intra-aortic balloon pumping; PCI, percutaneous coronary intervention.

### In-hospital mortality

The unadjusted in-hospital mortality rate of weekday daytime admissions was similar to that of patients admitted during the weekend night-time hours (Table 1). However, patients admitted during weekend daytime hours had a higher in-hospital mortality rate (7.6%) than those admitted during other times.

Table 3 shows the results of the multivariate analysis of in-hospital mortality after adjusting for the covariates. Admission during weekend daytime hours was significantly associated with higher in-hospital mortality (OR: 1.10, 95% CI: 1.03–1.19). Night-time admissions were not significantly associated with higher in-hospital mortality.

**Table 3:** Results of multivariable analysis of in-hospital mortality.

Variables	Adjusted odds ratio (95% CI)	*P*-value
Female	1.27 (1.19–1.35)	<0.001
Age, years		
18–60	Ref.	
61–70	1.50 (1.33–1.68)	<0.001
71–80	2.45 (2.19–2.74)	<0.001
≥81	5.81 (5.21–6.49)	<0.001
Ambulance use	1.31 (1.22–1.41)	<0.001
Killip classification on admission		
1	Ref.	
2	1.80 (1.63–1.98)	<0.001
3	5.26 (4.74–5.84)	<0.001
4	19.9 (18.2–21.7)	<0.001
Fatal arrhythmia	1.12 (1.01–1.23)	0.03
Atrial fibrillation	0.67 (0.60–0.76)	<0.001
Hypertension	0.33 (0.31–0.35)	<0.001
Hyperlipidemia	0.22 (0.20–0.24)	<0.001
Cerebrovascular disease	1.10 (0.98–1.25)	0.11
Diabetes	0.70 (0.66–0.76)	<0.001
Renal disease	1.62 (1.45–1.80)	<0.001
Chronic pulmonary disease	0.73 (0.60–0.89)	0.002
Old myocardial infarction	1.04 (0.84–1.30)	0.71
Number of cardiologists		
≥11	Ref.	
7–10	1.00 (0.97–1.36)	0.96
5–6	1.15 (0.97–1.36)	0.11
0–4	1.20 (1.03–1.40)	0.02
Admission		
Weekday daytime	Ref.	
Weekday night-time	0.94 (0.85–1.05)	0.30
Weekend daytime	1.10 (1.03–1.19)	0.006
Weekend night-time	1.05 (0.90–1.22)	0.56

Abbreviation: CI, confidence interval.

## Discussion

This multicenter study demonstrated that patients admitted to hospital during weekend daytime hours had a higher in-hospital mortality rate than patients admitted during other times, despite the similarly high rate of PCI performed on the day of admission in all 4 groups. In addition, weekend daytime admissions were significantly associated with higher in-hospital mortality even after adjusting for the covariates in the multivariable analysis. The prescription rates of guideline-based medication on the day of admission differed among the groups, with lower rates observed in daytime admissions than in night-time admissions.

Previous studies have reported that AMI patients admitted during off-hours have higher in-hospital mortality, but the evidence concerning this relationship is inconsistent [1,4,5,10–12,19,20]. Several studies have suggested that the higher mortality rate in AMI patients admitted during off-hours could be partially explained by the lower implementation rate of PCI [5,19], but this may have a relatively small effect on our analysis due to the higher PCI implementation rates for AMI patients in Japan (66–94%)[12,21] than in other countries (6–50%)[5,22]. In addition, although other studies have examined the relationship between off-hour admissions and higher in-hospital mortality in AMI patients, few studies have investigated the differences between office hour and off-hour admissions across weekdays and weekends [2,23–25]. Robinson et al. reported that the risk-adjusted outcomes in cardiac arrest patients were worse in both weekend daytime admissions (rate of return of spontaneous circulation >20 min: OR: 0.88 [95% CI: 0.81–0.95]; in-hospital survival: OR: 0.72 [95% CI: 0.64–0.80]) and night-time admissions (rate of return of spontaneous circulation >20 min: OR: 0.72 [95% CI: 0.68–0.76]; in-hospital survival: OR: 0.58 [95% CI: 0.54–0.63])[26]. Using a Danish medical registry, Vest-Hansen et al. reported that patients admitted during weekends tended to have higher 30-day mortality for 20 common medical conditions [25]. That study had also categorized patients into 4 groups: weekday office hours, weekday out-of-hours, weekend daytime hours, and weekend night-time hours. Their analysis found that AMI patients admitted during weekend office hours had the highest crude and standardized mortality rates relative to the other groups [25]. Those findings are similar to our results that weekend daytime admissions were associated with higher in-hospital mortality even after adjusting for various potential confounding factors. Due to the relative lack of studies on the effects of admission time across weekdays and weekends [2,23–25], our findings contribute to the further understanding of this aspect of care.

Although the mortality rates of patients who admitted during weekday daytime hours were not as high as patients admitted during weekend daytime hours. This disparity may have been affected by differences in the time from symptom onset to primary PCI. A meta-analysis reported that patients admitted during off-hours were less likely to receive PCI within 90 minutes than those admitted during regular hours [11] Furthermore, Japan has a relatively high number of consultations per capita among the Organisation for Economic Co-operation and Development member countries [27]. In addition, many clinics are closed on weekends, and the majority of patients would directly seek care at hospitals during this time. Accordingly, the high number of admissions and reduced staff numbers during weekend daytime hours may delay the time to treatment for AMI patients, thereby contributing to poorer prognoses.

This study also compared the prescription rates of medications recommended in Japanese guidelines for the treatment of AMI [28]. Our analysis found that the prescription rates of these medications on the day of admission were lower in weekend daytime than in night-time admissions. Moreover, these prescription rates were also lower in patients admitted during weekday daytime hours. Prescription rates may also have been affected by the hospitalization route. The clinic referral rates were higher in patients admitted during daytime hours than those admitted during night-time hours. During daytime hours, patients can seek treatment at nearby clinics instead of hospitals. Patients who are first diagnosed with AMI at a clinic may be administered the recommended medications before being transported to a hospital for advanced treatment. This could have partially contributed to the lower hospital prescription rates observed during daytime admissions.

Our study has several limitations that should be considered. First, the DPC administrative database does not provide detailed clinical information, including medical history or laboratory findings. As a consequence, we were unable to use several clinically relevant baseline variables such as door-to-balloon time. Due to the lack of these data, we had selected only patients with transmural AMI for the analysis. Moreover, we were able to use Killip class on admission as a severity index, which is rarely available in the administrative claims databases from other countries [29]. Studies have indicated that the addition of clinical variables to administrative databases improves their ability to be surrogate sources of clinical data [29,30].

Second, our study population was restricted to hospitals that voluntarily joined the DPC Study Group, which may have resulted in a degree of selection bias. However, this database contains approximately 8 million inpatient records per year, which account for more than half of all annual inpatient admissions to acute care hospitals in Japan. The large sample size and diverse characteristics of the hospitals may have reduced this selection bias.

Third, there are only 4 coding slots for comorbidities in the DPC administrative database. This limitation may result in an underestimation of the actual incidence of comorbidities. The inclusion of more coding slots may improve the accuracy of future analyses.

Fourth, we defined daytime hours as 6:00 AM to 9:59 PM, but 6:00 PM to 9:59 PM may traditionally be considered night-time hours. However, the definitions of daytime hours have varied substantially in previous studies [5,24–26]. Our definitions were based on those used in the hospital reimbursement system established by the national government. Due to their availability in the DPC database, we applied these definitions to our study.

Fifth, this study only included the Japanese population, and is therefore heavily influenced by the Japanese healthcare infrastructure. Accordingly, these findings may not be generalizable to other countries. Nevertheless, a government survey has found that the DPC database is representative of more than half of all Japanese acute care hospitals [31]. Our sample may therefore be considered representative of a large proportion of AMI patients in Japan.

## Conclusions

Patients with AMI admitted during weekend daytime hours had a higher in-hospital mortality rate than patients admitted during other times. Although more than 80% of the patients in this study received PCI on the day of admission in all 4 groups, patients admitted during daytime hours had lower prescription rates of guideline-based medications. Our findings indicate that weekend daytime admissions may be a potential weak point of the Japanese healthcare system that should be examined in greater detail.
