# Correction: Effects of night-time and weekend admissions on in-hospital mortality in acute myocardial infarction patients in Japan

**DOI:** 10.1371/journal.pone.0222125

**Published:** 2019-09-03

**Authors:** Seiko Mizuno, Susumu Kunisawa, Noriko Sasaki, Kiyohide Fushimi, Yuichi Imanaka

The first four authors, Seiko Mizuno, Susumu Kunisawa, Noriko Sasaki, and Kiyohide Fushimi, should not have been attributed equal contribution with the corresponding author, Yuichi Imanaka.
